# The Burden of Disease of Treatment‐Seeking Patients With a Cluster‐C Personality Disorder in the Netherlands; Quality of Life, Functioning, and Societal Costs

**DOI:** 10.1002/jclp.70109

**Published:** 2026-03-01

**Authors:** Iuno Z. Groot, Arnoud Arntz, Carlijn J. M. Wibbelink, Bas Verhoeven, Nathan R. Bachrach, Silvia M. A. A. Evers

**Affiliations:** ^1^ Department of Clinical Psychology University of Amsterdam Amsterdam the Netherlands; ^2^ Academic Center for Trauma and Personality (ACTP) Amsterdam the Netherlands; ^3^ Department of Medical and Clinical Psychology Tilburg University Tilburg the Netherlands; ^4^ Department of Personality Disorders GGZ‐Oost Brabant Helmond the Netherlands; ^5^ Department of Health Services Research, Care and Public Health Research Institute (CAPHRI) Maastricht University Maastricht the Netherlands; ^6^ Centre for Economic Evaluation, Trimbos Institute Utrecht the Netherlands

**Keywords:** burden of disease, cluster‐C personality disorders, cost‐off‐illness, quality of life, societal costs

## Abstract

Cluster‐C personality disorders (PDs) are common in the general population but often overlooked in scientific research and clinical practice. An important step to increase awareness for timely diagnosis and treatment of cluster‐C PDs is to investigate the burden of disease in terms of quality of life (QoL), daily functioning and the associated costs to society. In addition, a comparison between these patients and a control group can give insight into (1) the actual societal costs that are associated to cluster‐C PD problems and (2) potential societal savings to be made from therapeutic interventions. This study included a total of 375 treatment seeking individuals from 10 mental health sites for outpatient treatment in the Netherlands and 104 individuals without severe mental health issues from the general population. Both QoL (EQ‐5D‐5L and the MHQoL‐7D) and general functioning (WHODAS) were assessed as well as a comprehensive cost‐interview to measure relevant costs considering a societal perspective. Results indicate that individuals with a cluster‐C PD have severely impaired QoL and functioning compared to the control group. The annual costs associated with cluster‐C PD were estimated ranging from €27,355 to €60,454 per patient per year depending on the valuation method used. These costs equate 2.8–4.2 times more than those of control group. Looking at the subgroup in cluster‐C PD, no differences in QoL, functioning or societal costs were found between individuals with avoidant, dependent and obsessive‐compulsive PD. Overall, the findings advocate for more financial resources to study, prevent and treat cluster‐C PDs.

Cluster‐C personality disorders (PDs) are common in the general population (Volkert et al. 2018; Winsper et al. 2020) and highly prevalent (up to 30%) in clinical samples (Torgensen 2012). Individuals with a cluster‐C PD are characterized by heightened feelings of anxiousness, behavioral inhibition, and a strong need for control. These traits are often accompanied by impairments in interpersonal and self‐functioning, manifesting through patterns of avoidance, inhibition, and/or dependence (DSM; American Psychiatric Association 2013). While categorized within the same cluster, the three PDs in cluster‐C can differ substantially in their manifestation of symptoms and appearance. Obsessive‐Compulsive PD is the most common of the three (2.4%–4.1% of the general population; Winsper et al. 2020) and is characterized by being orderly, perfectionistic, and controlling. These patients often display a high degree of rigidity and stubbornness at the expense of efficiency and relaxation. Patients with an avoidant PD (1.9%–3.7% of the general population; Winsper et al. 2020) are described by social inhibition, feelings of inadequacy, and excessive sensitivity to negative judgment, which often leads to social withdrawal. The least prevalent PD (0.5%–1.3% of the general population) is dependent PD (Winsper et al. 2020). Patients with dependent PD have a great fear of abandonment and require permanent support and reassurance of others, they often feel incompetent and unable to make their own decisions and rely heavily on loved ones in daily life (American Psychiatric Association 2013).

To date, there has been little research on this group of PDs despite the high prevalence and high burden (however, progress is being made, with multiple promising intervention studies currently in progress, see: Daniëls et al. 2023; Groot et al. 2022; van den Heuvel et al. 2023). In addition, these disorders are often overlooked in clinical practice. A significant difficulty for individuals with a cluster‐C PD is late diagnosis. This delay is largely due to the internalizing nature of the symptoms, with interpersonal difficulties and high levels of avoidance often leading to social isolation (Bachrach and Arntz 2021). As a result, the disorders are less visible to others and can go unnoticed by clinicians for extended periods. When individuals do seek help, the focus of treatment is frequently on comorbid syndrome disorders, such as mood and anxiety disorders, which are common in this population (Hutsebaut et al. 2018). Such treatments don't address the full range of the mental health problems of the patient. Failure to recognize cluster‐C PDs may lead to undertreatment. Patients often return to mental healthcare in search of additional support, which highlights the need for more comprehensive and timely diagnostic assesment. An important step to increase awareness of the importance of timely diagnosis and treatment of cluster‐C PDs (both in research and clinical practice) is to investigate the burden of the disease in terms of quality of life (QoL), daily functioning and the associated costs to society.

## 1 Quality of Life and Functioning

Inherently to cluster‐C PDs are the prolonged and persistent patterns of interpersonal dysfunction and associated burden (American Psychiatric Association 2013). However, knowledge about how this dysfunction and burden manifests itself in cluster‐C PDs and what the influence is on daily life is scarce. In general, it is known that (cluster‐C) PDs are related to impaired psychosocial and physical functioning (Crawford et al. 2005; Jackson and Burgess 2004; Skodol et al. 2002). Lower functioning of individuals with a cluster‐C PD is also visible in a reduction of the amount and quality of friends and romantic relationships (Chen et al. 2006), lower employment rates and higher rates of psychotropic medication prescriptions (Coid et al. 2006). In addition, research shows a low QoL, with reduced physical health, mood, usual activities and relationships for individuals with a cluster‐C PD (Chen et al. 2006; Feenstra et al. 2012). This lowered QoL does not seem to differ from that of individuals with a cluster‐B PD (Kool et al. 2021). Consistently, a study comparing cluster‐C PD with cluster‐B PD showed no differences on work‐related, emotional and social functioning, challenging the assumption that cluster‐C represent less severe disorders (Massaal‐van der Ree et al. 2022). Research showed that PDs are associated with stronger impairments in subjective well‐being compared to syndrome disorders (Cramer et al. 2006).

When the PDs within cluster‐C are viewed separately, distinct differences with regard to well‐being emerge. For example, in terms of QoL, avoidant and dependent PD were found to be associated with poor QoL, whereas individuals with obsessive‐compulsive PD exhibited only limited impairment in a common population (Cramer et al. 2006). Similar results have been reported in a general population study of Grant et al. (2004), showing that obsessive‐compulsive PD was in general less disabling than other PD‐diagnoses such as avoidant and dependent PD. However, a study of a large clinical sample of treatment seeking patients showed opposite findings: obsessive‐compulsive, and not avoidant and dependent PD, predicted a lowered QoL (Soeteman, Hakkaart‐Van roijen, et al. 2008). Yet another clinical pilot study did not find any differences between the three cluster‐C diagnoses on QoL before treatment (Wibbelink et al. 2023). In terms of general functioning, two studies including treatment seeking individuals, found that individuals with obsessive‐compulsive PD had notable better general functioning before treatment compared to those with avoidant PD (Skodol et al. 2002; Wibbelink et al. 2023). Individuals with avoidant and dependent PD had comparable scores (Wibbelink et al. 2023).

These mixed results might be explained by the different sampling methods: while the clinical samples showed just as much ‐if not more‐ suffering in individuals with obsessive‐compulsive PD, general population studies indicated limited losses in subjective well‐being for obsessive‐compulsive PD. As Skodol et al. (2002) noted, treatment seeking individuals can be expected to be at a low point in life, influencing QoL negatively. When it comes to general functioning, obsessive‐compulsive PD may however occupy a distinct position. As some symptoms of obsessive‐compulsive PD (perfectionism, maintaining control, high productivity) may overlap with the current vision of “well‐functioning” in our society, problems in this regard might not reflect on general functioning measurements. However, this does not mean that individuals with obsessive‐compulsive PD suffer less. In addition, it has been pointed out that the current assessments commonly used to measure QoL may focus too much on physical discomfort, overlooking dimensions considered important for individuals with mental health issues (van Krugten et al. 2021). A newly developed questionnaire including clinically relevant dimensions like self‐image, independence and hopefulness about the future (van Krugten et al. 2021) is possibly more sensitive to measure wellbeing in this population. This current study will use different measures of QoL and functioning with the aim to picture a comprehensive view of well‐being of individuals with a cluster‐C PD.

## 2 Cost‐of‐Illness (COI)

An important approach to identify the burden of cluster‐C PD is through a COI analysis, which assesses the economic impact of specific disorders, offering insights not only into the total resources consumed due to the illness, but also into the primary cost components within healthcare, labor productivity losses and the strain on caregivers (Tarricone 2006). When conducted properly, COI studies can raise public awareness about overlooked disorders and encourage policymakers and health insurance companies to prioritize treatment and prevention strategies (Jo 2014; Rice 2000).

The few studies that have considered the costs of PDs show considerable societal costs, ranging approximately from €11,988 to €51,724 per person per year when adjusted for inflation to 2024 (Feenstra et al. 2012; Hastrup et al. 2019; Laurenssen et al. 2016; Soeteman, Hakkaart‐Van roijen, et al. 2008; Sveen et al. 2023; Wagner et al. 2022). The large variation in costs can be explained by differences in samples, contrasting methodological approaches and cost‐variations between nations. The chosen method for valuing productivity losses seems to be particularly significant (Sveen et al. 2023), which advocates for studies considering multiple valuing methods to enhance comparability. Nevertheless, these societal costs accompanying PDs are high, and outrange the costs of other mental health disorders like panic disorder, anxiety and mood disorders and seems comparable to costs for psychotic disorders and schizophrenia (Laurenssen et al. 2016; Sveen et al. 2023).

Of the limited studies that have been performed, very few investigated if differences in costs between (cluster‐C) PDs existed, showing mixed results (Feenstra et al. 2012; Soeteman, Hakkaart‐Van roijen, et al. 2008; Sveen et al. 2024). Results of Sveen et al. (2024) indicated no differences in overall societal costs between PDs but showed elevated productivity losses for individuals with avoidant PD along with those with borderline PD in comparison with other PDs. In contrast, Soeteman, Hakkaart‐Van roijen, et al. (2008) concluded that obsessive‐compulsive PD was a unique predictor of costs, relating to both higher productivity losses and higher overall costs. Feenstra et al. (2012) found no cost variations between types of PD. Moreover, while Soeteman, Hakkaart‐Van roijen, et al. (2008) and Feenstra et al. (2012) compared the costs of different PDs to a group of treatment seeking individuals without PD‐diagnoses, no comparisons have been made between the societal costs for cluster‐C PD with a control group of the general population. Such comparisons can however give great insight into potential societal savings that could be achieved through therapy.

## 3 This Study

In sum, despite the high prevalence of cluster‐C PDs, little research has investigated their personal and societal burden. Existing studies indicate impaired general functioning and reduced QoL among individuals with a cluster‐C PD. Upon a closer look, only a few clinical studies have investigated comparisons between the different types of cluster‐C diagnoses, with mixed results. In addition, only a few studies researched the costs accompanying (cluster‐C) PDs, but results remain difficult to compare due to differences in valuation methods. So far, no study compared societal costs of cluster‐C PD with a control group without severe mental health problems. This study therefore aims to: (1) Assess the burden of mental health (general functioning and QoL) and COI of treatment‐seeking patients with a cluster‐C PD in the Netherlands from a societal perspective in comparison with a control group; and in these data (2) Conduct subgroup analyses to study differences between avoidant, obsessive‐compulsive, and dependent PD.

## 4 Methods

### 4.1 Study Design

All clinical participants participated in a Randomized Controlled Trial (RCT) studying the (cost)effectiveness of (group)schema therapy (the QUEST‐CLC study; see Groot et al. 2022 for details). In short, this study took place at 10 mental health sites for outpatient treatment in the Netherlands: PsyQ Zaandam, PsyQ Amsterdam, PsyQ Utrecht, PsyQ Rotterdam, Emergis, GGZ‐OB Helmond, GGZ‐OB, Boxmeer, GGZ‐OB Oss, IPGGZ Veendam, and IPGGZ Groningen. This study was approved by the Ethics Committee of the University of Amsterdam (nr. 2020‐CP‐12948) and is registered at the Netherlands Trial Register; NL9209. All patients received extensive information about the offered treatments and the accompanying assessments that would take place and signed an informed consent. After a screening procedure to check for in‐ and exclusion criteria, a baseline assessment was conducted. This assessment is the first of in total five assessments over a 2‐year period. All interviews were administered by well‐trained research assistants on site. Only data that is collected during this first baseline assessment was used for the current study. A nonpatient comparison group was formed by 104 individuals from the general population recruited through convenience and referral sampling.

### 4.2 Sample Characteristics

A total of 375 individuals with a primary cluster‐C PD were included. Between April 2021 and June 2023 patients were approached to participate in this study when a primary cluster‐C PD was suspected. Inclusion criteria consisted of (1) a primary diagnosis of a cluster‐C PD (avoidant PD, obsessive‐compulsive PD or dependent PD) as was determined by well‐trained clinicians and confirmed with the Structured Clinical Interview for DSM‐5 Personality Disorders (SCID‐P; Arntz et al. 2017); (2) be of at least 18 years of age; (3) be proficient in the Dutch language; and (4) motivated and available to participate in one of the three forms of therapy for 1 year and complete the related assessments. Individuals were excluded if they had (1) an acute risk of suicidality (as determined by a clinician); (2) received a form of schema therapy within a year prior to inclusion.

For the comparison group 104 individuals were recruited in November and December 2023 from the general population through convenience and referral sampling, using social media and word‐of‐mouth.^1^ Of these individuals, 12 withdrew before the assessment, resulting in 92 participants completing the interviews. Exclusion criteria consisted of (1) a diagnosis of a personality disorder; (2) severe cognitive complaints in the last 6 months; or (3) having received specialized mental healthcare in the previous 6 months. To match the demographics of the study participants, efforts were made to recruit individuals who (1) were female (aiming for a gender ratio of 40% male to 60% female); (2) were aged between 20 and 50 years old; (3) were distributed equally across various educational levels. Participants completed the same interviews (cost‐interview and WHODAS) as the study participants via telephone and filled out the questionnaires online. Participants were compensated with a €7.50 gift card.

## 5 Materials

### 5.1 Quality of Life

To obtain an estimation of QoL, we used two self‐report measures: the 5 level EuroQol 5D (EQ‐5D‐5L; (The EuroQol group 1990) and the Mental Health Quality of Life Questionnaire (MHQoL‐7D; van Krugten et al. 2021). The EQ‐5D‐5L is the most used QoL measurement within economic evaluations and records QoL in five dimensions: mobility, self‐care, usual activities, pain/discomfort, and anxiety/depression. Response options for each dimension are scored on five levels ranging from no problems (1) to extreme problems or unable to (5). From these, the Dutch utility values (as described in Versteegh et al. 2016) were calculated resulting in utilities ranging from −0.446 (worst outcome) to 1 (best possible QoL). The EQ‐5D‐5L exhibits excellent psychometric properties across different populations and settings (Feng et al. 2021). In the current study the Cronbach's alpha was satisfactory (*α* = 0.71).

In addition, the MHQoL‐7D was administered as it was specifically developed to measure QoL in individuals with subclinical and clinical mental health problems. This questionnaire records seven domains of QoL; self‐image, independence, mood, relationships, daily activity, physical health and future, which can be answered on a scale from 0 (*very dissatisfied*) to 3 (*very satisfied*). The index value ranges from −0.741 (worst state) to 1 (best state; van Krugten et al. 2024). The MHQoL‐7D was able to distinguish between mental healthcare service users and members of the general population and has a high internal consistency (*α* = 0.85; van Krugten et al. 2021). In our sample, high reliability was found *α* = 0.83.

### 5.2 General Functioning

The World Health Organization Disability Assessment Schedule 2.0 (WHODAS 2.0; World Health Organization 2000) was included to measure objective general functioning. The WHODAS 2.0 interview addresses six dimensions of functioning: cognition, mobility, self‐care, getting along with people, life activities at work and home and participation in society. It contains 36 items which can be answered on a 5‐point Likert scale ranging from “no difficulties” to “extreme difficulties.” The complex scoring method was used to compute total scores; higher scores indicate higher dysfunction. The WHODAS 2.0 has a very high internal consistency in recent studies (*α* = 0.91 to 0.97; Üstün et al. 2010) and a satisfactory reliability in our sample *α* = 0.78.

### 5.3 Cost Measurement

The costs are measured from a societal perspective, considering all expenses associated with cluster‐C PD, regardless of who incurs them. This included healthcare costs, costs faced by patients and their support system, productivity costs and financial impacts on sectors outside of healthcare, such as education (Drummond et al. 2015). The authors developed a cost interview to determine relevant costs for this specific population. This included, for example, a comprehensive assessment of health costs (due to potential higher somatization within this population), focus on psychopharmaceuticals (because of high comorbidity with symptomatic disorders) and additional questions on other costs that could be related to a lowered mental health, e.g. costs for impulsive shopping, ordering food because one is not capable of cooking, binge‐eating, tickets that were bought but never used, or excessive telephone‐use. The interview was based on existing inventories such as the Trimbos/iMTA questionnaire for Costs associated with Psychiatric Illness (TIC‐P; Bouwmans et al. 2013) and was extended to fit the population. The interview consisted of 57 items (with additional follow‐up questions), covering resource use over the last 18 weeks. An example item is*:* “In the past 18 weeks, have you called on (or been helped by) family, relatives, friends and/or others because you could not be alone, needed someone to care for you, to look after you, or to accompany you to a care provider?”. Costs were clustered into three categories; (1) Healthcare‐related costs, which incorporates all costs related to mental and somatic healthcare including alternative care; (2) Patient and support system costs, including travel expenses, informal care, over the counter medication, alcohol and drugs expenditures and other costs that could be related to a lowered mental health (see above); (3) Productivity losses of (unpaid) work, study, and domestic activities. To estimate all costs, a bottom‐up approach is used; the amount of service used is multiplied by the relevant unit cost and summed up to form the total costs (Drummond et al. 2015).

### 5.4 Valuation

For the valuation of all measured resources, our first preference was to adopt reference prices of the Dutch manual of costs analysis in healthcare research (Hakkaart‐van Roijen et al. 2024). For specific healthcare professions that were not available in the manual, the cost price of comparable occupations (e.g., consults at an addiction care clinic were valued at the same costs for specialized outpatient treatment in a mental health facility) or adopted cost prices from the Dutch Healthcare Authority (NZa) were used. For psychopharmaceuticals, four medication types were defined: anti‐depressants, sedatives, antipsychotic, and mood stabilizers. For each category, medication within this type were identified and an average price was calculated based on the Daily Defined Dosage (DDD). We presumed this to be chronic medication and thus applied costs for daily use including prescription costs every 90 days (following Hakkaart‐ van Roijen et al. 2024). For alcohol consumption, consumer prices were obtained from both supermarkets and bar/restaurants (Eurostat 2016; Statistics Netherlands 2019). The price was then calculated on the ratio of consumptions at home versus at a bar/restaurant based on data involving a comparable population (Wibbelink et al. 2025). For cigarettes and drugs expenditures and for the other expenses related to a lowered mental health, the study used costs as reported by the participant. Travel costs were based on the average distance to a medical facility along with parking costs (Hakkaart‐van Roijen et al. 2024). For facilities with unknown distances, the distance to a physiotherapist was adopted. For admissions with overnight stay where it was not clear how often individuals were traveling, a conservative approach was being taken, calculating one time travel costs. For informal care, volunteer work and productivity losses in domestic activities shadow prices were used. Hours missed in educational activities were valued using standard prices per educational level with a maximum of 940 h per year (Hakkaart‐van Roijen et al. 2024). When educational level was unknown, an average of the standard prices was applied. All valuations of resources can be found in Table S7, within the Supporting Information.

To estimate productivity losses for paid labor, we used the friction cost (FC) method as base case. The FC method assumes that employees will be replaced within a limited time frame and includes only the costs incurred during the friction period (i.e., the moment of replacement). In the Netherlands, the present friction period is estimated to be 115 calendar days (Hakkaart‐ van Roijen et al. 2024). Cost prices are expressed in 2024 euros. Costs made in 2020 till 2023 were corrected using the consumer price index (CPI). Because the cost interview had a recall period of 18 weeks (±4 months), all costs were multiplied by 2.89 to estimate yearly costs.

### 5.5 Sensitivity Analyses

As valuation of costs is always based on certain assumptions, it is useful to explore different scenarios for the most uncertain estimates (Schnitzler et al. 2023). Within our analyses, we determined three uncertainties of which we calculated the costs in an alternative manner. First, to estimate productivity losses for paid labor, we used two alternative approaches, as the FC method has been criticized for being too strict for estimating costs in chronic disorders like PDs (van Asselt et al. 2008). Within the Human Capital Approach (HCA), productivity losses were not corrected for the friction period, resulting in an estimation of all absent hours of work within 1 year. With the third approach, HCA‐extended, productivity losses were based on all potentially lost earnings under the assumption that there is a full use of labor. We choose to estimate full use of labor for all participants; for those with or without a job and/or with disability benefits or welfare we compared productivity with the average working hours of the Dutch population when gender and age are considered (Statline 2024). For participants who studied we compared productivity with average working hours for students in the Netherlands (Statistics Netherlands 2023).

Second, a sensitivity analysis was performed regarding informal care, where the maximum hours spent per day was limited to 16 h per day, to take hours of sleep into account. And lastly, as costs for prescribed medication (apart from psychopharmaceuticals) and over‐the‐counter medication were originally not incorporated in our cost interview at baseline but were added at the following assessments of our RCT (see for further details Groot et al. 2022), we did not include these in the main analyses. In a sensitivity analyses, expenses at baseline were estimated based on the ratio of costs for psychopharmaceutic to costs for other medication found in the follow‐up assessment, assuming this ratio would be stable over different time points. These medications were valued based on lowest price available for the DDD on the Dutch Pharmacotherapeutic Compass in July 2024 (National Healt Care Institute ‐ Netherlands 2024). A pharmacist and psychiatrist helped to unravel vague descriptions of certain medications. For most over the counter medication we followed the participants' reported costs, except for commonly used painkillers and some medication that were difficult to account for costs per use, like nasal spray and cough syrup. Here we opted the costs for the cheapest available medication of that type in a well‐known drugstore in the Netherlands. All medication prices were excluding VAT.

### 5.6 Analysis

All analyses were performed in SPSS (version 28). Missing data was < 1% on the overall dataset. Variables with the highest missing data were costs of cigarettes and drugs, with 2.71% and 2.51% missing data, respectively. The Dutch manual of costs analyses described that more straightforward approaches as mean imputation are considered acceptable when missing data is less than 5% (Hakkaart‐van Roijen et al. 2024). Therefore, missing data was based on personal average in following assessments, or if not available, on the average of the total sample. Only unrealistic values were corrected, but outliers were kept as they were expected to represent true values. First, the cluster‐C PD group was compared to the comparison group on both QoL, functioning and societal costs using bootstrapped independent *t*‐tests. Second, subgroup analyses were performed to examine differences between the three cluster‐C PD‐diagnoses using bootstrapped independent *t*‐tests. Due to the small sample size, pairwise comparisons were only conducted between the avoidant PD and the other two diagnoses. To correct the skewness in the distribution of the data (which was the case for all costs and for the QoL and functioning measurements), Bias‐corrected and accelerated (BCa) bootstrapping with a total of 1000 simulations were performed. To correct for multiple comparisons, we applied the False Discovery Rate (FDR) method (Benjamini and Hochberg 1995) for all results with an original *p* value of 0.05.

## 6 Results

The demographics for the cluster‐C PD group and the control group are shown in Table 1. Group differences were found for employment status, showing fewer individuals with a job within the cluster‐C PD group. The groups did not differ significantly on age, gender or educational level.

**Table 1:** Demographical information and QoL and functioning scores of the cluster‐C PD and the comparison group.

	Total cluster‐C PD (*N* = 375)	Comparison group (*N* = 86)	
	*M* (SD)	*M* (SD)	Analysis
Age	37.23 (11.16)	34.69 (12.67)	*t* = 1.85, *p* = 0.065
Educational level^a^	4.62 (1.73)	4.43 (1.56)	*t* = 1.02, *p* = 0.310
	*n* (%)	*n* (%)	
Gender			Fisher's test = 1.05, *p* = 0.59
Female	247 (65.9%)	58 (67.4%)	
Male	126 (33.6%)	27 (31.4%)	
Other	2 (0.5%)	1 (1.2%)	
Employment status			Fisher's test = 43.51, ***p *< 0.001**
Working	171 (45.60%)	58 (67.4%)	
Sick leave	53 (14.13%)	3 (3.5%)	
Disability	68 (18.13%)	3 (3.5%)	
Welfare	26 (6.93%)	3 (3.5%)	
Unemployed	23 (6.13%)	0 (0%)	
Student	34 (9.07%)	17 (19.8%)	
Other^b^	0 (0%)	2 (2.3%)	

^a^ Educational level was categorized using the International Standard Classification of Education, 2011 version (UNESCO, 2012).

^b^ Other consisted of individuals who were retired or who received survivor's pension.

### 6.1 Quality of Life and General Functioning

Due to non‐normal distribution of the data in the comparison group, independent *t*‐tests with BCa‐adjustments were performed to examine differences in QoL‐ and functioning measurements between the cluster‐C and comparison group. Significant differences were observed between the two groups on all measures (see Table 2). Individuals with cluster‐C PD showed lower scores on both the QoL‐measures (MHQoL‐7D and EQ‐5D‐5L) and higher scores on the WHODAS, indicating greater dysfunction with large effect sizes (Cohen's *d* ranging from 1.48 to 1.67).

**Table 2:** Mean scores (standard deviation) of the QoL and functioning measures for the cluster‐C PD and the comparison group.

	Cluster‐C PD group (*N* = 375)		Comparison group (*N* = 86)		Analysis	
	*M* (SD)	Boot *M*	*M* (SD)	Boot *M*		
MHQoL‐7D index^a^	0.35 (0.35)	0.35	0.88 (0.14)	0.88	*p* = 0.001	*d* = 1.48
EQ‐5D‐5L index	0.59 (0.23)	0.59	0.91 (0.12)	0.91	*p* = 0.001	*d* = 1.67
WHODAS	35.60 (15.04)	35.60	12.87 (10.51)	12.13	*p* = 0.002	*d* = 1.59

Abbreviations: Boot M = Bootstrapped mean, EQ‐5D‐5L = EuroQol EQ‐5D, MHQoL‐7D = Mental Health Quality of Life Questionnaire, WHODAS = The World Health Organization Disability Assessment Schedule II.

^a^ One individual of the cluster‐C PD group had missing data on the MHQoL‐7D resulting in *N* = 374 for the cluster‐C group.

### 6.2 Societal Costs

The total societal costs for individuals with a cluster‐C PD were found to be €27,355 per person per year, which significantly differed from the comparison group with €7,421 per person per year. Bootstrapping analyses revealed differences between the two groups across the three cost categories: with a small effect size for patient and support system costs, a medium effect size for productivity losses and a large effect size for healthcare costs. Resource use and societal costs per group, and between group comparisons, are shown in Table 3.

**Table 3:** Recourse use and societal costs for individuals with a cluster‐C PD and the comparison group.

	Cluster‐C PD (*N* = 375)				Comparison group (*N* = 86)					
	Resource use	Societal costs (€)			Resource use	Societal costs (€)				
	*M*	*M*	Boot *M*	Boot CI	*M*	*M*	Boot *M*	Boot CI	Analysis	
Healthcare costs	—	*7275.48*	*7258.14*	*6446.20–8157.29*	—	*1072.72*	*1071.29*	*690.19–1464.85*	***p*** < **0.001**	*d* = 0.86
*Mental Health Institution*										
Outpatient treatment^a^	27.12	4102.11	4095.10	3644.93–4571.50	0.87	113.23	117.72	11.21–247.74	***p*** < **0.001**	*d* = 0.94
Admission with overnight stay	0.61	213.22	212.22	39.83–466.64	0.00	0.00	0.00	0.00–0.00	*p* = 0.210	*d* = 0.10
Outreaching care	2.41	339.17	336.07	149.19–567.52	0.00	0.00	0.00	0.00–0.00	***p*** = **0.018**	*d* = 0.19
Crisis service (in minutes)	60.58	144.79	144.86	56.76–266.77	0.00	0.00	0.00	0.00–0.00	*p* = 0.143	*d* = 0.14
Outpatient treatment private practice^b^	0.32	41.36	41.58	14.59–77.46	0.24	30.06	31.27	0.00–83.39	*p* = 0.782	*d* = 0.04
Social worker	1.79	244.24	244.77	148.53–348‐78	0.00	0.00	0.00	0.00–0.00	***p*** = **0.003**	*d* = 0.27
Occupational health services^c^	4.68	604.24	602.95	474.53–756.21	0.34	43.37	43.59	11.00–84.98	***p*** < **0.001**	*d* = 0.18
Mental health nurse practitioner	0.95	21.17	20.93	13.45–29.69	0.35	7.88	7.81	0.00–18.61	*p* = 0.058	*d* = 0.19
General practitioner	4.65	153.89	154.43	136.22–172.37	2.92	96.65	96.47	69.62–151.80	***p*** = **0.002**	*d* = 0.31
*General hospital*										
Outpatient treatment	2.22	286.24	283.19	218.52–370.33	1.51	194.34	194.31	107.05–292.50	*p* = 0.132	*d* = 0.14
Day treatment	0.15	55.30	54.39	16.93–105.85	0.07	24.11	23.99	0.00– 61.04	*p* = 0.319	*d* = 0.07
Overnight stay	0.32	223.25	222.99	60.50–458.32	0.34	231.78	226.52	44.79–494.28	*p* = 0.959	*d* = −0.00
Emergency care^d^	0.11	29.81	29.85	15.07–46.23	0.17	46.42	45.62	10.31–91.85	*p* = 0.451	*d* = −0.10
Paramedical treatment	7.39	290.97	288.94	224.24–369.49	3.33	130.98	132.52	69.30–200.91	***p*** = **0.003**	*d* = 0.24
Alternative therapy^e^	1.03	75.53	75.50	42.25–113.08	0.64	47.05	46.02	13.16–93.14	*p* = 0.335	*d* = 0.09
Other^f^	4.48	197.66	197.14	97.37–313.97	1.01	71.39	70.64	0.00–199.00	*p* = 0.172	*d* = 0.12
Psychopharmaceuticals	—	252.53	252.73	217.53–287.59	—	35.44	34.79	4.79–77.29	***p*** < **0.001**	*d* = 0.68
Patient and support system costs	—	*4832.53*	*4783.01*	*3558.74–6533.78*	—	*1618.47*	*1609.07*	*861.56–2597.63*	***p*** = **0.006**	*d* = 0.25
Informal care	160.58	3232.47	3190.19	2014.51–4830.55	28.87	581.08	574.79	88.20–1441.70	***p*** = **0.003**	*d* = 0.21
Travel costs	—	326.28	325.67	296.99–356.88	—	56.86	56.72	41.46–75.02	***p*** < **0.001**	*d* = 1.02
Alcohol	—	208.42	208.20	3173.77–245.62	—	480.24	481.94	380.79–599.63	***p*** = **0.002**	*d* = −0.67
Tabaco	—	342.59	342.31	245.25–438.41	—	201.77	203.37	114.69–294.21	*p* = 0.045^h^	*d* = 0.17
Drugs	—	58.41	58.38	32.13–89.03	—	80.21	79.80	31.49–142.66	*p* = 0.519	*d* = −0.07
Other expenses^g^	—	664.37	658.27	510.98–850.81	—	218.31	215.40	29.27–565.09	*p* = 0.089	*d* = 0.29
Productivity losses	—	*15354.28*	*15314.37*	*13601.20–17263.23*	—	*4720.10*	*4305.89*	*2924.4737–6585.9545*	***p*** < **0.001**	*d* = 0.65
Paid work	222.22	9490.95	9462.42	8006.59–11018.84	87.61	3741.99	3769.80	2218.09–5345.4790	***p*** < **0.001**	*d* = 0.40
Absenteeism	134.86	5759.91	5722.65	4644.72–7161.3309	46.44	1992.85	2012.60	977.1021–3156.04	***p*** < **0.001**	*d* = 0.33
Presenteeism	87.38	3731.04	3739.77	2986.73–4506.06	40.95	1749.15	1757.18	908.66–2800.48	***p*** = **0.005**	*d* = 0.25
Education	35.94	876.34	868.12	506.84–1298.95	9.61	188.81	189.50	43.86–368.57	***p*** = **0.007**	*d* = 0.19
Losses in domestic activities	247.74	4986.99	4983.82	4241.33–5790.03	39.21	789.30	769.6115	332.57–1351.90	***p*** < **0.001**	*d* = 0.58
Total costs	—	27462.29	27355.52	24814.83–30474.13	—	7411.29	7421.07	5384.17–9730.41	***p*** **<** **0.001**	*d* = 0.84

*Note: p*‐values in bold indicate statistical significance at the 0.05 level, with FDR correction applied to the different cost categories.

Abbreviations: Boot CI = Bootstrapped confidence interval, Boot M = Bootstrapped mean.

^a^ Psychologist, psychiatrist, psychiatric nurse or creative arts therapist, either basic or specialized treatment.

^b^ Including basic or specialized treatment.

^c^ Including company doctor and work‐integration programs

^d^ Including emergency department of the hospital and ambulance

^e^ Including acupuncture, reiki, coaches, homeopathist, and so forth.

^f^ Rehabilitation care, outpatient addiction care, municipal public health service, and home care.

^g^ Other costs that could be related to a lowered mental health, for example costs for impulsive shopping, ordering food because one is not capable of cooking, binge‐eating, tickets that were bought but never used, or excessive telephone‐use.

^h^ This comparison was not significant under the FDR corrected significance level of *p *= 0.0313. All other effects remained significant under the FDR correction.

Upon a closer look, significant cost differences between the two groups were found for the following healthcare related resources: outpatient treatment in mental health institution, outreaching care, social worker, occupational health services, general practitioner, mental health nurse practitioner, paramedical treatment, and the use of psychopharmaceuticals. No differences were found for costs related to hospital visits, crisis service, outpatient treatment in private practice or alternative therapy. The main cost driver within healthcare‐related expenses for the cluster‐C PD group was outpatient treatment (56.4%), followed by occupational health services (8.3%). For the control group, the highest contributor was overnight stay at hospitals (21.6%).

Within the category of patient and support system costs the group of cluster‐C PD incurred higher costs on informat care and travel expenses compared to the control group. No differences were observed for (recreational) drugs use, tabaco and other expenses, whereas alcohol‐related costs were higher in the control group. Costs for the cluster‐C PD group stemmed mostly from informal care (66.9%), a trend which was also observed in the control group to a lesser extent (35.9%).

On all aspects of productivity losses, individuals with a cluster‐C PD had higher costs than the control group. For both groups the majority of the costs were related to absenteeism in paid labor: 61.8% for the cluster‐C PD versus 79.3% for the control group. Although a high percentage of the productivity losses was also explained by being less productive (presenteeism): 37.5% in cluster‐C PD and 42.2% for the control group.

### 6.3 Sensitivity Analyses

#### 6.3.1 Productivity Losses

As previously discussed, the use of the FC method has been criticized for possibly underestimating productivity losses. For this reason, and to enhance comparability with international studies, we also reported productivity losses and their impact on total societal costs under the HCA and the extended version of the HCA (see Table 4). Under the assumptions of both the HCA and HCA‐extended, bootstrapped results showed significantly higher costs for the cluster‐C PD group in comparison to the comparison group. When considering all lost productive hours (HCA), total societal costs increased substantially for individuals with a cluster‐C PD to €31,651 per year. When lost potential (HCA‐extended) was alco accounted for, costs rose to €60,454 per year. For the control group the total costs increased to €7,605 (HCA) and €21,993 (HCA‐extended) respectively.

**Table 4:** Losses in paid work and total societal costs under the friction cost method (FC), human capital approach (HCA) and human capital approach‐extended (HCA extended) for individuals in the cluster‐C PD and comparison group.

	Cluster‐C PD (*N* = 375)			Comparison group (*N* = 86)			Analysis	
	*M*	Boot *M*	Boot CI	*M*	Boot *M*	Boot CI		
*Losses in paid work*								
FC	9490.95	9462.42	8006.59–11018.84	3741.99	3769.80	2218.09–5345.4790	***p*** < **0.001**	*d* = 0.40
HCA	13804.97	13758.42	11619.79–16305.49	3932.71	3967.31	2341.17–5563.47	***p*** < **0.001**	*d* = 0.47
HCA‐extended	42540.23	42561.01	39646.65–45581.54	18257.73	18316.80	14588.84–22183.19	***p*** < **0.001**	*d* = 0.88
*Total societal costs*								
FC	27462.29	27355.52	24814.83–30474.13	7411.29	7421.07	5384.17–9730.41	***p*** < **0.001**	*d* = 0.84
HCA	31776.31	31651.52	28421.57–35346.48	7602.00	7605.28	5564.12–10078.02	***p*** < **0.001**	*d* = 0.83
HCA‐extended	60511.57	60454.11	56260.55–64852.71	21927.02	21993.79	17715.96– 26638.22	***p*** < **0.001**	*d* = 1.06

Abbreviations: Boot CI = bootstrapped confidence interval, Boot M = bootstrapped mean, FC = friction cost method, HCA = human capital approach.

#### 6.3.2 Informal Care

A second sensitivity analyses was performed for the costs related to informal care, in which the maximum hours spend on informal care were limited to 16 h per day. The average costs of informal care did not change for the control group, but the average costs for the cluster‐C PD groups reduced to M = €2897.99 (Boot M = 2867.72). This was not significantly different from the original informal care costs, *p* = 0.094, *d* = 0.10. The difference in informal care costs was still significant between the control and the cluster‐C group, *p* = 0.002, *d* = 0.22.

#### 6.3.3 Medication

To estimate medication costs at baseline, the ratio of psychopharmaceuticals to other medication observed at follow‐up (*n* = 152) was applied. The estimated annual costs per person were €106.30 for prescriped medication and €24.31 for over‐the‐counter medication.

### 6.4 Subgroup Analyses

Bootstrapped, pair‐wise comparisons were conducted to test if there were differences in the QoL and general functioning for avoidant, obsessive‐compulsive and dependent PD. Demographics of the subgroups are shown in Table S8, within the Supporting Information. Test statistics on the QoL and functioning measures are shown in Table 5. Results show that there were no differences for the subgroups on the EQ‐5D‐5L and WHODAS. On the MHQoL‐7D however, the dependent PD group scored significantly higher than the avoidant PD group. However, since the dependent PD group is very small, these results should be interpreted with caution.

**Table 5:** QoL and functioning scores for the group of individuals with avoidant, obsessive‐compulsive and dependent personality disorder.

	Avoidant PD (*n* = 287)		Obsessive‐compulsive PD (*n* = 77)		Dependent PD (*n* = 11)		Between group comparison^a^
	*M* b	Boot CI	*M*	Boot CI	*M*	Boot CI	
MHQoL‐7D^c^	0.32	0.29–0.36	0.41	0.33–0.48	0.50	0.34–0.67	A vs OC, *p* = 0.067, *d* = −0.24 A vs. D, ***p*** **= 0.035**, *d* = −0.51
EQ‐5D‐5L	0.59	0.56–0.62	0.62	0.56–0.67	0.64	0.55–0.72	A vs. OC, *p* = 0.356, *d* = −0.12 A vs. D^2^, *p *= 0.251, *d* = −0.22
WHODAS	35.96	34.28–37.68	34.73	31.06–38.39	32.27	25.48–39.29	A vs. OC, *p *= 0.552, *d* = 0.08 A vs. D^2^, *p* = 0.347, *d* = 0.25

Abbreviations: Boot CI = bootstrapped confidence interval, Boot M = bootstrapped mean, EQ‐5D‐5L = EuroQol EQ‐5D, MHQoL‐7D = Mental Health Quality of Life Questionnaire, WHODAS = The World Health Organization Disability Assessment Schedule II.

^a^ Group comparisons were conducted using bootstrapped independent *t*‐test, comparing individuals in the avoidant PD group with obsessive‐compulsive PD and dependent PD separately.

^b^ Bootstrapped means did not differ from the original means and are thus not displayed. Only exception is for the dependent PD group: The bootstrapped mean for the WHODAS was boot M = 32.44.

^c^ One individual of the avoidant PD group had missing data on the MHQoL‐7D resulting in *n* = 286 for the group of individuals with avoidant PD.

For the societal costs, pair‐wise comparisons were made between the avoidant PD and the other two diagnoses. Bootstrapped independent *t*‐test were conducted to test if the subgroups differed on the three main costs categories and on total societal costs. Because the subgroup of individuals with a dependent PD was very small, these results are meant to be descriptive. As can be seen in Table 6, results did not differ significantly between the subgroups. Non‐parametric tests showed similar results.

**Table 6:** Societal costs for the avoidant, obsessive‐compulsive and dependent PD.

	Avoidant PD (*n* = 287)			Obsessive‐compulsive PD (*n* = 77)			Dependent PD (*n* = 11)			Between group comparison^a^
	*M*	Boot *M*	Boot CI	*M*	Boot *M*	Boot CI	*M*	Boot *M*	Boot CI	
Healthcare costs	7042.60	7023.86	6296.33–7912.34	7514.61	7543.21	5623.50–9536.34	11677.73	11793.19	5616.49–18152.75	A vs. OC, *p* = 0.671, *d* = −0.06 A vs. D,^b^ *p* = 0.171, *d* = −0.62
Patient and support system costs	3982.06	3979.69	2944.18–5327.07	6001.70	6190.26	3381.24–9092.27	18837.84	19271.54	2646.60–45903.45	A vs. OC, *p* = 0.315, *d* = −0.17 A vs. D,^b^ *p* = 0. 424, *d* = −1.09
Productivity losses	14755.66	14805.32	12986.95–16415.08	15977.26	16048.51	12374.74–19383.76	26611.75	26355.59	7707.18–55834.02	A vs. OC, *p* = 0.549, *d* = −0.07 A vs. D,^b^ *p* = 0.426, *d* = −0.66
Total costs	25780.32	25808.87	23406.94–28421.63	29493.57	3151.07	24024.66–34683.79	57127.33	57420.30	23216.70–95889.72	A vs. OC, *p* = 0.270, *d* = −0.16 A vs. D,^b^ *p* = 0.150, *d* = −1.25

Abbreviations: Boot CI = bootstrapped confidence interval, Boot M = bootstrapped mean.

^a^ Group comparisons were conducted using bootstrapped independent *t*‐test, comparing individuals in the Avoidant PD group with Obsessive‐Compulsive PD and Dependent PD separately.

^b^ Comparison between the Avoidant PD and Dependent PD were conducted with stratified bootstrapped independent *t*‐tests.

## 7 Discussion

The objective of this study was to describe the burden of disease in terms of QoL, functioning, and societal costs for treatment seeking individuals with a cluster‐C PD compared to a control group. In addition, subgroup analyses were performed to study differences between avoidant, obsessive‐compulsive and dependent PD. Results showed that individuals with a cluster‐C PD have impaired QoL and general functioning as compared to the control group. Looking at the cluster‐C subgroups, no differences in QoL and functioning were found between individuals with avoidant, dependent and obsessive‐compulsive PD. The annual costs associated with a cluster‐C PD were estimated ranging from €27,355 to €60,454 per patient per year depending on the valuation method used. These costs equate 2.8–4.2 times more than those of the comparison group without severe mental health issues.

Individuals with a cluster‐C PD showed severely impaired scores on both QoL‐measures, with a value set of 0.59 for the EQ‐5D‐5L and of 0.35 for the MHQoL‐7D which differed greatly from the comparison group with values of 0.91 and 0.88 respectively. Differences in scores on the two assessment methods indicate that the more recently developed MhQoL‐7D might (as intended) indeed be more susceptible to measure QoL in a population with mental health problems than the EQ‐5D‐5L. Although there are no norms available yet for the MHQoL‐7D, scores for our sample were slightly higher than those of borderline PD patients (0.24; Wibbelink et al. 2025). Scores on the EQ‐5D‐5L were slightly higher, but comparable to the 0.55 that Feenstra et al. (2012) and the 0.56 that Soeteman, Hakkaart‐Van roijen, et al. (2008) reported within a similar treatment seeking population of individuals with diverse PDs in the Netherlands. The EQ‐5D‐5L‐scores of the cluster‐C PD group are considerably lower than that of the average Dutch population (0.87; Versteegh et al. 2016) and of a population with depression and anxiety disorders of 0.73 (Franklin et al. 2021). In addition, functioning was severely impaired in the cluster‐C PD group in comparison to the control group. Scores found on the WHODAS, measuring general dysfunctioning were comparable to those found in a similar cluster‐C PD population (ranging from 30.99 to 39.27; Wibbelink et al. 2023) and higher (indicating more disability) than in patients with depressive disorder (27.14; Chwastiak and Von Korff 2003).

When examining QoL and general functioning at the subgroup level, the only significant difference observed was a higher MHQoL‐7D score for individuals with dependent PD compared to those with avoidant PD. Due to the limited sample size of the dependent PD group, these findings should be interpreted with caution. Replication of the results is necessary before drawing further conclusions or making broader generalizations. No other significant subgroup differences were found. This is contradictory to earlier studies, indicating that obsessive‐compulsive PD is associated with less impairment compared to the other PD diagnoses (Cramer et al. 2006; Grant et al. 2004; Skodol et al. 2002). These discrepancies might be explained by different sampling manner; while these earlier studies included the general population, or a population that was already in outpatient treatment, our study solely focused on a clinical sample seeking treatment. Skodol et al. (2002) suggested that treatment seeking individuals can be expected to be at a low point in life, influencing functioning negatively. However, this does not account for the discrepancy with Wibbelink et al. (2023), who studied a similar cluster‐C PD population (although with a small obsessive‐compulsive PD sample of *n* = 19) and found better general functioning in the obsessive‐compulsive PD group compared to those with avoidant PD. One possible explanation may lie in the fact that neither study considered the number of PD‐diagnoses, while Soeteman, Hakkaart‐Van roijen, et al. (2008) showed this to be a stronger predictor than specific diagnoses.

The low QoL and functioning scores for the cluster‐C group illustrate the high psychological distress experienced by this group of patients. So far, no longitudinal studies have yet been conducted on how QoL and functioning evolves in time for individuals with a cluster‐C PD. However, without proper treatment, cluster‐C symptoms are known to remain relatively stable or worsen over time (Wu and Francois 2022), which will not be beneficial for improving QoL and functioning. On the positive side, studies have shown multiple therapeutic interventions to be effective in improving QoL and general functioning in cluster‐C PD samples (Bamelis et al. 2014; Bartak et al. 2010; Wibbelink et al. 2023). Bamelis et al. (2014) showed improvements on both QoL and functioning with large effect sizes for different forms of therapy, with schema therapy showing the most progression on general functioning in comparison to clarification therapy and treatment as usual. Wibbelink et al. (2023) demonstrated improvements on both QoL and functioning following group schema therapy. Here, individuals with a dependent PD seemed to benefit the most; they showed significantly more improvement in general functioning in comparison to individuals with avoidant and obsessive‐compulsive PD. Taken together, present and previous research highlights the need to ensure that this group receives appropriate treatment, as individuals with cluster C PDs can benefit greatly from it.

The annual societal costs accompanying cluster‐C PDs were estimated at €27,355 when using the FC method, €31,651 for the HCA and €60,454 when the HCA‐extended was applied. The costs differed significantly from the comparison group whose cost were valued at €7,421, €7,605, and €21,993 respectively. No cost differences were found between the three cluster‐C PD diagnoses. Compared to earlier studies, the annual costs for individuals with a cluster‐C PD were higher than those reported by Bamelis et al. (2015); approximately €19,030 (in 2024 prices) for a predominantly cluster‐C sample using the FC method. When compared with samples from other PD groups, remarkably, costs of the cluster‐C group remained only slightly lower to those found in a borderline PD group, which ranged from €35,038 under the FC‐method to €68,873 under the HCA‐extended method (Wibbelink et al. 2025), highlighting the significant disease burden of cluster‐C PDs. In relation to broader PD samples, the annual costs reported here exceed those reported by Soeteman, Verheul, et al. (2008) (adjusted for inflation to approximately €16,805), who employed the FC method to estimate productivity losses across various PDs. This discrepancy may be partly attributed to the present study's more comprehensive assessment of patient and support system‐related costs. Conversely, the costs observed in this study are slightly lower than those reported by Sveen et al. (2023) (approximately €50,974 in 2024 prices), who used the HCA method. Differences may be partially explained by Sveen's direct inclusion of welfare costs, whereas the HCA‐extended method employed here incorporated welfare indirectly by valuing unused potential. Overall, the findings indicate that cluster‐C PDs are associated with substantial economic costs for both patients and society, comparable to those of borderline PD, despite the latter often being perceived as a more severe PD condition.

The huge differences in costs found in this study between the different valuation approaches for productivity losses (FC, HCA, and HCA extended) underline the importance of choosing the appropriate method, or using multiple approaches, when it comes to COI research. We used the FC method as base case as it is recommended by the Dutch manual (Hakkaart‐van Roijen et al. 2024) and to improve comparability with other studies performed in the Netherlands. However, as already argued, the FC‐method might be too rigid for more chronic disorders like PDs, which can be of additional significance within our relatively short recall‐period. For instance, the FC‐method does not include costs for sick leave or disability after the friction period ends, ignoring individuals who are absent for the entire duration of the recall period. As 32% of our cluster‐C sample was on sick leave or received a disability benefit, this is a considerable cost driver, which is reflected in the high costs under the HCA‐approach. In addition, there are many individuals with a cluster‐C PD who do have a job but work less hours than their colleagues because of their lowered capacity (Coid et al. 2006). The substantial increase in costs between the HCA and HCA‐extended approaches indicates that the cluster‐C PD group indeed experiences a significant loss of potential, also surpassing that of the control group. Our advice is not to underestimate the persistent nature of PDs and their associated costs.

The comparison between our sample of individuals with cluster‐C PD and a control group offers insights into the potential societal cost savings associated with therapeutic interventions. Specifically, this comparison enables an evaluation of whether the observed resource use and associated costs are exclusively attributable to PD‐related factors or if they also occur within the general population, a topic that has received limited attention in prior research. For instance, certain healthcare expenses, productivity losses, and other economic burdens may also arise in what is described as a “healthy population,” due to somatic complaints or accidents. By subtracting the costs incurred by the control group from those attributed to the cluster‐C PD group, we estimate that the potential societal savings resulting from therapy to be approximately €19,934 to €38,551 per person per year, depending on method of valuation. Empirical data on the actual expenses associated with interventions for cluster‐C PD and the cost‐effectiveness of these treatments remain limited. Bamelis et al. (2015) estimated the annual costs of individual schema therapy and clarification‐oriented psychotherapy for cluster‐C PD at €6,476 and €8,569, respectively (adjusted to 2024 prices), with schema therapy demonstrating greater cost‐effectiveness. Soeteman et al. (2011) identified short‐term inpatient psychotherapy as the most cost‐effective treatment for Cluster‐C PD, with estimated annual costs of €36,843. The QUEST‐CLC study aims to further contribute to this body of research by investigating the cost‐effectiveness of both group and individual schema therapy in this population, as potential cost and effect differences between group ST and individual ST are still unknown (Groot et al. 2022).

To answer the question of where the greatest societal savings can be made, we can examine the relatively high cost contributors. These contributors may also serve as important points for clinicians to address in treatment, as they could facilitate improved societal integration for patients. For example, productivity losses constituted roughly 56% of the total costs in the cluster‐C PD sample. Notable is the large number of hours missed by being less productive (presenteeism) and the accompanying costs, highlighting the substantial struggles these patients face in functioning at work in daily life. As these struggles are also visible in relatively high costs for occupational health services, lower employment rates and higher dysfunctioning scores on the WHODAS, systematically addressing work‐related difficulties during treatment and setting goals with regard to work‐rehabilitation might be beneficial for this population and may reduce societal cost. Another significant cost is informal care, which indicates the considerable burden on the support system of this population. Including partners, family members and/or friends in therapeutic interventions can improve visibility of informal care and seek solutions to lighten the weight for the support system.

This study involves some sampling considerations that warrant further discussion. First, individuals in our cluster‐C PD sample with acute suicidality were excluded. This possibly influenced the results for the QoL, functioning and costs measures, for instance, due to decreased use of crisis services, reduced productivity losses and informal care and so forth. Second, although cluster‐C PD are known to be highly comorbid with mood‐ and anxiety disorders (estimated between 30% and 50% with syndrome disorders; Hutsebaut et al. 2018) we did not control for these comorbidities in our analyses. While it is likely that comorbid disorders contribute to lower QoL, impaired functioning, and increased costs, our aim was to describe the overall burden of disease in the cluster‐C population, including the comorbidities that are inherently part of these disorders. Future research could further disentangle the sources of burden by examining the impact of disorders and comorbidities as predictors of disease burden.

Third, we applied the current classification system for PD diagnoses, which relies on a categorical system. This categorical model is still the definitive standard, as it is recommended by the DSM‐5 and remains widely adopted in clinical practice. However, there is increasing concern among researchers and clinicians about its limitations. An alternative model has been proposed, which includes a continuous scale to express severity of the PD. This new model also omits certain PD diagnoses, although avoidant and obsessive‐compulsive PDs are retained. Consequently, the findings of this study remain relevant, even under potentially different methods of classification. Future research could consider examining whether disorder severity (e.g., number of criteria) is predictive of disease burden when utilizing the new model.

Fourth, some of the data ‐collection for the cluster‐C PD group was conducted during the COVID‐pandemic, which could possibly result in deviating productivity losses and healthcare costs, with perhaps differential effects in the two groups. Fifth, the sample size of individuals with a dependent PD included in this study was small and results should be interpreted with caution. The limited inclusion of individuals with dependent PD in this study lacks a definitive explanation. Based on prevalence data, a higher representation of individuals with this diagnosis would typically be expected within a treatment‐seeking sample. One possible explanation is that dependent PD is often associated with feminine characteristics (Disney 2013), which may lead to it being more frequently overlooked in men. Indeed, we did not include male participants with dependent PD in our sample. Additionally, the overlap in criteria with other PDs, specifically avoidant PD (Gude et al. 2004), which may present more prominent symptoms, could result in these other disorders being identified as the primary diagnosis. Also, the prevalence of this PD seems to reduce in Western countries, due to women emancipation and changes in rearing practices.

Another limitation of our study is that our control group may not be fully representative of the general population. To compare with earlier studies, the societal costs of our control group were at least twice as high as those reported by Hastrup et al. (2019), which came to about €3,120 when inflation is considered. The observed differences can be partially attributed to Hastrup's use of a less comprehensive cost assessment, which excludes certain factors such as patient and family costs. However, our reported costs remain higher in comparison. On the other hand, when compared to the general Dutch population under the age of 65, healthcare costs were lower than expected (€5,319 in 2024 prices; (National Institute for Public Health and the Environment 2019). Our sample was matched to certain characteristics (gender, age and level of education) of the PD population. This aligns with our aim of assessing actual PD‐related costs and comparing them to the baseline costs of a comparable group within the general population. However, this means it is not a random sample from the general population and should not be interpreted as such. Discrepancies within the few studies with control groups indicate the need for future research to gather more data from the general population.

Furthermore, COI‐studies face various challenges and considerations that require thoughtful examination. For example, although our study included a comprehensive cost‐interview, some aspects that could contribute to societal costs were omitted. Police contact was not assessed since no elevated costs for this specific population were expected. However, this is a contributor to societal costs and future studies could include this. An unclear but potentially relevant factor for this population is spending more time on (un)paid work or domestic tasks due to perfectionism and high demands. Similarly, difficulties in cooperating with colleagues or becoming a burden at work are challenging to assess but worth pursuing, especially for individuals with an obsessive‐compulsive PD (Ettner et al. 2011). In addition, our study tried to assess volunteer work, however this item was wildly misunderstood due to the vague formulation and consequently was not included in the main analyses. To make an estimation of productivity losses in volunteering, we can look at the costs for the control group, which were about €56 per year and that of a population of borderline PD of about €152 per year (Wibbelink et al. 2025). Future studies are advised to take these factors into account when assessing COI.

Another challenge lies in accurately valuing certain resources. In informal care, for example, quantifying exact caregiving hours is difficult, as routine activities can often overlap with care (Brouwer et al. 1999). On the other hand, it is hard to measure other elements of caregiving; loved ones can experience fatigue and mental health problems themselves and have less leisure time to recharge. Other costs that could have been estimated incorrectly include costs for alcohol consumption and alternative therapy. For these resources we relied on standard prices, however, money spent on these resources can vary greatly between individuals. Finally, the projected costs for psychopharmaceuticals may represent an overestimation as the calculations assumed consistent daily use. However, variations in usage patterns, such as‐needed administration, could lead to lower actual expenditures. Future studies can take these considerations into account.

To conclude, this study showed a high level of suffering for individuals with a cluster‐C PD, visible in a severely lowered QoL and general functioning. In addition, a cluster‐C PD is accompanied with considerable costs for society which greatly exceed those of a matched comparison group. QoL, functioning and costs did not differ between avoidant, dependent and obsessive‐compulsive PD. All in all, these results advocate for more financial resources to study, prevent and treat cluster‐C PDs. The results underscore that when there is evidence suggesting a cluster‐C PD, investing additional time in the diagnostic process is essential to ensure an appropriate treatment. Increased funding for such diagnostic and therapeutic efforts is justified and needed to lower societal costs and improve well‐being.

## Conflicts of Interest

The authors declare no conflicts of interest.

## Supporting information

Supplementary materials_Burden of disease.

## Endnotes

1 Note that the data of the comparison group is also published in (Wibbelink et al. 2025), although differences can appear due to differences in costs and valuation methods.
