# Supplementary material for: The Burden of Disease of Treatment‐Seeking Patients With a Cluster‐C Personality Disorder in the Netherlands; Quality of Life, Functioning, and Societal Costs
Source: J Clin Psychol. 2026 Mar 1;82(5):775–89. doi: 10.1002/jclp.70109 (PMC13064880; doi:10.1002/jclp.70109)
Supplement: Supplementary file 1 — Supplementary materials_Burden of disease. [file JCLP-82-775-s001.docx]

**Supplementary materials**

**Table 7***Unit of Quantity and Value of all Resources*

| **Resource** | **Unit of Quantity** | **Price in euro** |
| --- | --- | --- |
| **Health care costs** |  |  |
| *Mental health institution* |  |  |
| Specialized outpatient treatment^a^ (individual session) | Consult | 143.51 |
| Specialized outpatient treatment^a^ (group session^b^) | Consult | 71.76 |
| Basic outpatient treatment^a^ (individual session) | Consult | 129.59 |
| Basic outpatient treatment^a^ (group session^b^) | Consult | 64.80 |
| Crisis service | Minute | 2.39 |
| Outreaching care | Consult | 180.61 |
| Admission with overnight stay^c^ | Day/night | 350.22 |
| Day treatment | Day | 182.22 |
| Outpatient treatment in private mental health practice^d^ | Consult | 127.78 |
| Social worker | Consult | 136.02 |
| Mental health Nurse practitioner (POH-GGZ) | Consult | 22.33 |
| Self-help group | Consult | 10.07 |
| Peer support services | Session |  |
| Work integration program | Consult |  |
| Company doctor | Consult | 128.52 |
| General practitioner | Consult | 33.06 |
| *General hospital* |  |  |
| Outpatient treatment | Consult | 128.52 |
| Day treatment | Day | 358.79 |
| Overnight stay | Day/night | 689.72 |
| Ambulance | Consult | 565.49 |
| Emergency care | Consult | 276.32 |
| Rehabilitation care | Consult | 532.29 |
| Paramedical treatment | Consult | 39.37 |
| Alternative treatments^e^ | Consult | 73.69 |
| *Home care* |  |  |
| Household assistance | Consult | 67.90 |
| Care assistance | Consult | 94.48 |
| Medical assistance | Consult | 113.14 |
| *Psychopharmaceutic* |  |  |
| Antidepressants | DDD | 0.75 |
| Sedatives | DDD | 0.19 |
| Antipsychotic | DDD | 1.91 |
| Mood stabilizers | DDD | 0.50 |
| Other prescribed medication | DDD | Various |
|  |  |  |
| **Patient and support system costs** |  |  |
| Informal care | Hours | 20,13 |
| Travel costs |  |  |
| *Out-of-pocket cost* |  |  |
| Beer^f^ | Glass | 1.31 |
| Wine^f^ | Glass | 1.50 |
| Liquor^f^ | Glass | 1.66 |
| Tabaco |  | As reported |
| Drugs |  | As reported |
| Over the counter medication |  | As reported |
| Other expenses |  | As reported |
|  |  |  |
| **Productivity Losses** |  |  |
| *Paid work* |  |  |
| Absenteeism | Hour | 42.71 |
| Presenteeism^g^ | Hour | 42.71 |
| Education^h^ | Hour | Various^h^ |
| Unpaid work | Hour | 20.13 |
| Losses in domestic activities | Hour | 20.13 |

Note. All costs prices are reported in 2024 euros.

^a^ Psychologist, psychiatrist, psychiatric nurse of creative arts therapist in a mental health institution.

^b^ Based on a double consult, eight patients and two care providers per group.

^c^ Also used for admissions related to substance abuse.

^d^ Psychologist, psychotherapist or psychiatrist in either basic or specialized treatment.

^e^ Based on the average prices provided by various professional associations of alternative care providers. For professions lacking a professional association, averages were derived from reported costs within a Borderline population dataset (Wibbelink et al., 2025).

^f^ Weighted averages of the prices for alcohol bought in a store vs bought at a bar (Statistics Netherlands, 2019; Eurostat, 2016) Weighting is based on data of a population of Borderline PD (Wibbelink et al., 2025)

^g^ Based on the percentage that individuals indicated to be unproductive.

^h^ Based on level of education; Secondary vocational education (€22.24), higher professional education (€18.19), university (€33.88) and other or education level unknown an average of (€24.77; Hakkaart- van Roijen et al., 2024)

**Table 8**
*Means (Standard Deviations) of the Demographic for the Group of Individuals with Avoidant, Obsessive-compulsive and Dependent Personality Disorder*

|  | **Avoidant PD**  **(*n* = 287)** | **Obsessive-Compulsive PD (*n* = 77)** | **Dependent PD**  **(*n* = 11)** | **analysis** |
| --- | --- | --- | --- | --- |
|  | *M (SD)* | *M (SD)* | *M (SD)* |  |
| Age | 36.57 (11.21) | 39.10 (10.45) | 41.18 (13.17) | *F* = 2.29, *p* = .103 |
| Educational level | 4.48 (1.75) | 5.16 (1.53) | 4.72 (1.80) | *F* = 4.82, ***p* = .009^a^** |
|  |  |  |  |  |
|  | *n* (%) | *n* (%) | *n* (%) |  |
| Gender |  |  |  |  |
| Female | 193 (67.2%) | 43 (55.8%) | 11 (100%) |  |
| Male | 93 (32.4%) | 33 (42.9%) | - |  |
| Other | 1 (0.3%) | 1 (1.3% | - |  |
| Employment status |  |  |  | Fisher’s test = 12.29, ***p* = .012** |
| Working | 135 (47.04%) | 31 (40.26%) | 5 (45.45%) |  |
| Sick leave | 35 (12.20%) | 16 (20.78%) | 2 (18.18%) |  |
| Disability | 54 (18.82%) | 14 (18.18%) | 0 (0%) |  |
| Welfare | 21 (7.32%) | 5 (6.50%) | 0 (0%) |  |
| Unemployed | 14 (4.88%) | 5 (6.50%) | 4 (36.36%) |  |
| Student | 28 (9.76%) | 6 (7.79%) | 0 (0%) |  |
| Other | 0 (0%) | 0 (0%) | 0 (0%) |  |

*Note*.
^a^Post-hoc analyses revealed group differences between the avoidant and obsessive-compulsive PD, with higher levels of education for the group of obsessive-compulsive disorder.
